# Immunization with Plant-Derived Multimeric H5 Hemagglutinins Protect Chicken against Highly Pathogenic Avian Influenza Virus H5N1

**DOI:** 10.3390/vaccines8040593

**Published:** 2020-10-09

**Authors:** Hoang Trong Phan, Van Thi Pham, Thuong Thi Ho, Ngoc Bich Pham, Ha Hoang Chu, Trang Huyen Vu, Elsayed M. Abdelwhab, David Scheibner, Thomas C. Mettenleiter, Tran Xuan Hanh, Armin Meister, Ulrike Gresch, Udo Conrad

**Affiliations:** 1 Leibniz Institute of Plant Genetics and Crop Plant Research (IPK), D-06466 Seeland OT Gatersleben, Germany; armin.meister@t-online.de (A.M.); ulgresch@hotmail.de (U.G.); 2 Institute of Biotechnology (IBT), Vietnam Academy of Science and Technology (VAST), Hanoi 100000, Vietnam; phamthivan1103@gmail.com (V.T.P.); htthuong.biotech@gmail.com (T.T.H.); pbngoc@ibt.ac.vn (N.B.P.); chuhoangha@ibt.ac.vn (H.H.C.); vuhuyentrang.ibt@gmail.com (T.H.V.); 3 Faculty of Biotechnology, Graduate University of Science and Technology (GUST), VAST, Hanoi 10000, Vietnam; 4 Institute of Molecular Virology and Cell Biology, Friedrich-Loeffler-Institut, Federal Research Institute for Animal Health, D-17493 Greifswald-Insel Riems, Germany; Sayed.abdel-whab@fli.de (E.M.A.); David.Scheibner@fli.de (D.S.); ThomasC.Mettenleiter@fli.de (T.C.M.); 5 National Veterinary Joint Stock Company (NAVETCO), 29 Nguyen Dinh Chieu, Dist 1, Ho Chi Minh City 700000, Vietnam; tranxuanhanhnavetco1@gmail.com

**Keywords:** avian flu, vaccination, plant-expressed hemagglutinin (H5), oligomerization, immunization, crude extracts, protective immune response, chicken

## Abstract

Since 2003, H5N1 highly pathogenic avian influenza viruses (HPAIV) have not only caused outbreaks in poultry but were also transmitted to humans with high mortality rates. Vaccination is an efficient and economical means of increasing immunity against infections to decrease the shedding of infectious agents in immunized animals and to reduce the probability of further infections. Subunit vaccines from plants are the focus of modern vaccine developments. In this study, plant-made hemagglutinin (H5) trimers were purified from transiently transformed *N. benthamiana* plants. All chickens immunized with purified H5 trimers were fully protected against the severe HPAIV H5N1 challenge. We further developed a proof-of-principle approach by using disulfide bonds, homoantiparallel peptides or homodimer proteins to combine H5 trimers leading to production of H5 oligomers. Mice vaccinated with crude leaf extracts containing H5 oligomers induced neutralizing antibodies better than those induced by crude leaf extracts containing trimers. As a major result, eleven out of twelve chickens (92%) immunized with adjuvanted H5 oligomer crude extracts were protected from lethal disease while nine out of twelve chickens (75%) vaccinated with adjuvanted H5 trimer crude extracts survived. The solid protective immune response achieved by immunization with crude extracts and the stability of the oligomers form the basis for the development of inexpensive protective veterinary vaccines.

## 1. Introduction

The vaccination of humans and animals to protect against infectious diseases is an efficient preventive measure [1]. Vaccines used in veterinary medicine, particularly those for application in food-producing animals, have to be cost effective and should increase the health of animals and significantly reduce the use of other compounds [2]. Zoonotic pathogens that can infect animals as well as humans, such as avian flu [3] caused by orthomyxoviruses, are common sources of emerging infectious diseases. Wild waterfowl populations provide a large and highly mobile reservoir for type A influenza viruses (AIVs). Avian flu caused strong damage to the poultry and the tourist industry in south-east Asia [4]. Highly pathogenic avian flu viruses were responsible for outbreaks in South Korea, China, Japan, USA, Canada and many European countries [5]. Influenza viruses constantly change via recombination to express different combinations of the major viral antigens hemagglutinin and neuraminidase and via mutagenesis [6,7] to escape the immune response of the host [8].

Vaccination is an efficient and economical means of increasing immunity against infections to decrease the shedding of infectious agents in immunized animals and to reduce the probability of further infections [9], thus achieving herd immunity. Subunit vaccines from plants, which exhibit benefits such as low production cost, easy scale up, low infrastructure cost, high stability and a long half-life, are the focus of modern vaccine developments [10]. However, the subunit vaccines induced generally low immunogenicity against influenza [11]. It has been demonstrated that the immunogenicity of influenza hemagglutinins depend on their oligomeric nature. High molecular weight hemagglutinins (oligomers) induced stronger antibody responses than trimers and monomers [12,13]. In addition, oligomeric levels of influenza hemagglutinin depend on cell sources [12], or on expressed hemagglutinin fragments [13]. Generic methods that can produce oligomeric subunit vaccines are not available. Recently, Phan and co-workers have shown general methods to produce oligomeric antigens both in vivo [14] and in vitro [15]. However, oligomeric antigens produced by both methods have not yet been evaluated by viral challenge experiments. Here, we developed a proof-of-principle approach using plant-expressed hemagglutinin for the protection of chickens against AIV by maintaining the trimeric structure of hemagglutinin combined with oligomerization, leading to successful immunization with crude leaf extracts. The solid protective immune response achieved by immunization with crude extracts and the stability of the oligomers form the basis for the development of inexpensive protective veterinary vaccines, especially suitable for application in developing countries and suiting demands for highly effective vaccines, simple and severe production strategies and efficient and applicable ways and methods for distribution and use.

## 2. Materials and Methods

### 2.1. Construction of Plant Expression Vectors

**Figure 1:**
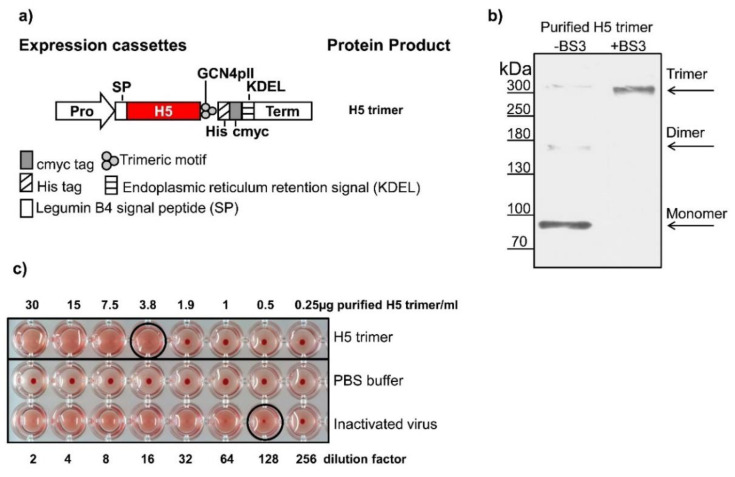
Expression and characterization of plant-derived H5 trimers. (**a**) Plant expression cassette. The ectodomain of hemagglutinin was C-terminally fused to the GCN4pII motif for trimerization, a c-Myc tag for downstream detection by Western blotting and a His tag for purification by IMAC. The legumin B4 signal peptide (SP) and the KDEL motif caused ER retention [17]. Pro: cauliflower mosaic virus 35S ubiquitous promoter; term: cauliflower mosaic virus 35S terminator. (**b**) Oligomeric state of the H5 trimer confirmed by a crosslinking reaction. Purified H5 proteins were supplemented with the BS3 crosslinker at 5 mM or left untreated and visualized by Western blotting with an anti-c-Myc monoclonal antibody. (**c**) Functional characterization of purified H5 trimers by the hemagglutination assay. Two-fold serial dilutions of purified H5 trimers were mixed with the given amounts of chicken red blood cells, and hemagglutination was recorded. Circles indicate hemagglutination titers.

The DNA hemagglutinin sequences of A/duck/Viet Nam/TG24-01/2005 (H5N1) (hereafter referred to as H5-TG), A/duck/Viet Nam/HT2/2014 (H5N1) (hereafter referred to as H5HT) strains and others were codon-optimized for expression in *N. benthamiana*, and synthesized (GENECUST EUROPE, Luxembourg). DNA sequences coding for H5-TG and H5HT trimers were cloned into pRTRA-35S-H5pII [16] containing the trimeric motif GCN4pII at the BamHI and Bsp120I sites to form recombinant vectors designated pRTRA-H5-TGpII (Figure 1a) and pRTRA-H5HTpII. Recombinant proteins contained the KDEL motif for the endoplasmic reticulum (ER) retention [17]. DNA sequences coding for the connectors were introduced after the trimeric motif GCN4pII to multimerize H5-TG trimers. These connectors are tail piece elements at the c-terminus of mouse IgM antibody chains that form disulfide bonds via its cysteine residues [18] (Figure 3 and Tables S1 and S2), homoantiparallel peptides [19,20,21] (HAP) (Figure 3 and Tables S1 and S2), and homodimer proteins [22,23] (HDP) (Figure 3 and Tables S1 and S2). The resulting pRTRA vectors were designated as pRTRA-H5-TP, pRTRA-H5-HDP, and pRTRA-H5-HAP, respectively. All inserted sequences in pRTRA vectors were verified by sequencing. Expression cassettes in pRTRA vectors were digested by HindIII enzyme and cloned into the pCB301 shuttle vector. The pCB301 shuttle vectors were transformed into the agrobacterium strain pGV2260. Expression cassettes of the recombinant oligomeric H5 proteins were presented in Figure 3.

### 2.2. Transient Protein Expression in N. Benthamiana Plants

Recombinant hemagglutinins were transiently expressed in *N. benthamiana* leaves by using the agrobacterium vacuum infiltration method as described by Phan and Conrad [24]. Recombinant agrobacteria containing H5 expression vectors (Figure 1 and Figure 3) as well as agrobacteria harboring the plant HcPro expression vector (suppressor of gene silencing as an enhancer) were separately cultured in Luria-Bertani (LB) medium containing antibiotics (50 µg/mL kanamycin, 50 µg/mL carbenicillin and 50 µg/mL rifampicin) overnight at 28 °C and 140 rpm. The pre-cultures were put in a new LB culture with appropriate antibiotics. After 24 h of cultivation, bacteria were harvested (centrifugation at 4000× *g*, 30 min, 4 °C) and resuspended in infiltration buffer (10 mM 2-(N-morpholino) ethanesulfonic acid (MES), 10 mM MgSO_4_, pH 5.6). Both agrobacterial cultures were combined and diluted in infiltration buffer to a final OD600 of 1.0. *N. benthamiana* plants (six to eight weeks old) were infiltrated by completely submerging each plant in an agrobacterium-containing cup standing inside a desiccator. Vacuum was applied for 2 min and then quickly released. The plants were then grown in the greenhouse at 21 °C with 16 h light/8 h dark conditions. Five days post vacuum infiltration, the infiltrated leaves were taken and stored at −80 °C.

### 2.3. Total Soluble Protein Extraction

Twenty grams of the infiltrated leaves were ground in liquid nitrogen and homogenized in phosphate buffered saline (PBS) (1:3 ratio; mass:volume) with a commercial blender. The extracts were centrifuged at 16,200× *g*, 30 min, at 4 °C. Protein concentrations of clarified plant extracts were determined by Bradford assay.

H5 contents in crude plant extracts were semi-quantified by Western blotting. Known concentrations of the anti-tumour necrosis factor nanobody-elastin-like polypeptide (aTNF-ELP) standard protein [25] for the c-Myc-tag were used to compare blot signal intensities.

### 2.4. SDS-PAGE and Western Blotting

Proteins were prepared in an sodium dodecyl sulfate (SDS) sample buffer (final concentration: 31.25 mM Tris-HCl, pH 6.8; 12.5% (*v*/*v*) glycerol; 1% (*w*/*v*) SDS; 0.005% (*w*/*v*) bromophenol blue; 2.5% (*v*/*v*) β-mercaptoethanol), cooked 95 °C for 5 min. Treated proteins were separated on 10% SDS gels at reducing conditions. After electrophoresis, proteins were transferred into nitrocellulose membranes. The blotted membranes were incubated with monoclonal anti-c-Myc antibodies, and subsequently incubated with horseradish peroxidase-linked sheep anti-mouse IgG antibodies [26]. Enhanced chemiluminescent (ECL) detection reagents were applied to the membranes. Chemiluminescent signals were captured on films.

To detect H5-specific chicken antibodies raised against plant extracts containing H5 oligomer-tailpiece (TP) and H5 trimer, 700 ng of the size exclusion chromatography (SEC)-purified H5 trimer proteins were separated on 6 lanes of a reducing SDS–PAGE gel (10% polyacrylamide) and transferred to a nitrocellulose membrane. The membrane was blocked with a 5% (*w*/*v*) fat-free milk powder dissolved in a PBS buffer for 2 h. Each lane of the membrane was separated by cutting and incubated at room temperature for 90 min with a 1:20 dilution of a mixture of sera from ten chickens of each group after the second immunization. The membranes were incubated with a 1:2000 dilution of an alkaline-conjugated goat anti-chicken IgY secondary antibody for 1 h at room temperature. Specific signals were detected by incubating membranes with 3,3–diaminobenzidine (DAB, Thermo Scientific Pierce) dissolved in 0.05 M tris-HCl and 0.04% hydrogen peroxide for 10 min in the dark.

### 2.5. Protein Purification by Immobilized Metal Affinity Chromatography (IMAC)

Infiltrated leaves were ground in liquid nitrogen, and resuspended in 50 mM tris buffer, pH 8.0. The extracts were centrifuged (75,600× *g*, 30 min, 4 °C). The clarified extracts were mixed with Ni Sepharose 6 Fast Flow (GE Healthcare, Uppsala, Sweden) (washed twice with water before) for 30 min at 4 °C. The mixture was applied to a chromatography column. The column was extensively washed (50 mM NaH2PO4, 300 mM NaCl, 30 mM imidazole, pH 8.0). Recombinant proteins were eluted from the column with elution buffer (50 mM NaH2PO4, 300 mM NaCl, 125 mM imidazole, pH 8.0), added to dialysis bags, concentrated in PEG 6000 and dialyzed against PBS.

### 2.6. Size Exclusion Chromatography

Thirty-four micrograms of purified IMAC H5 proteins/0.5 mL were loaded onto a SuperoseTM 6 increase 10/300GL column (GE Healthcare, Uppsala, Sweden). The column was fed with 48 mL of PBS buffer at 0.5 mL/min flow rate. Fractions containing five hundred microliters were collected for hemagglutination test and Western blot analyses. The high molecular weight standard proteins (44 kDa to 669 kDa) were loaded onto the column to estimate the molecular weight of the proteins of interest.

Affinity-purified trimeric hemagglutinin (A/duck/Vietnam/TG24-01/2005) was further purified via the SuperoseTM 6 increase 10/300 GL column with starting concentrations of 1.25 mg in 0.5 mL. The resulting SEC purified H5 trimer was coated to plates for indirect ELISA.

### 2.7. Mouse Immunization

Western blotting was used to semi-quantitatively estimate H5 contents in plant extracts. Plant crude extracts were prepared to contain equal H5 contents (100 ng of either H5 oligomers or H5 trimers). Wild-type (WT) plant extract containing equal total soluble protein amount as H5 oligomer plant extracts was included in mouse experiments as a negative control group. Plant lysates were mixed with the Emulsigen^®^-D adjuvant (MVP Technologies, 4805 G Street, Omaha, NE, USA) at 20% final concentration. Twelve male C57/Bl6/J mice per group (Charles River Laboratories GmbH) were subcutaneously vaccinated with Emulsigen^®^-D adjuvant-formulated plant extracts at days 0, and boosted at days 14 and 28. Mouse sera were collected one week after the second and third immunizations for hemagglutination inhibition and ELISA tests.

The mouse animal experiments (“Testung von oligomeren Vogelgrippevakzinekandidaten aus Pflanzen in Mäusen“, Aktenzeichen 42502-3-864 IPK) were approved by the Landesverwaltungsamt Sachsen-Anhalt, Halle/Saale, Referat Verbraucherschutz, Veterinärangelegenheiten and by the Landkreis Harz, Amt für Veterinärwesen und Lebensmittelüberwachung, Halberstadt. All animals received humane care according to the requirements of the German Animal Welfare Act, §8 Abs. 1.

### 2.8. Hemagglutination Test and Hemagglutination Inhibition Assay

The hemagglutination test was performed with 1% chicken erythrocytes [24]. The highest dilution of antigens or viruses causing complete hemagglutination was defined as one hemagglutination unit (HAU). The hemagglutination inhibition (HI) assay was done with mouse sera using 4 HAU of the inactivated rg A/swan/Germany/R65/2006 (H5N1) virus. The amino acid sequence distance matrix between the inactivated rg A/swan/Germany/R65/2006 (H5N1) strain and other three strains is presented in Table S2. Two-fold serial dilutions of H5 antisera were prepared across the row of V shape plates. Four HAU of the inactivated rg A/swan/Germany/R65/2006 (H5N1) virus in 25 µL PBS was added to every well of the plates. Plates were shaken at 25 °C for 30 min. A 1% suspension of chicken erythrocytes was added. HI titer was read after the plates were kept at 25 °C for 30 min. The HI titer is defined as the reciprocal of the highest dilution of serum that could completely inhibit hemagglutination.

To analyze HI titers from chicken sera, inactivated A/Chicken/DL/NAVET_0292/2013 (H5N1) viruses were used.

### 2.9. Indirect ELISA

Each 100 µL per well of 1 µg/mL of IMAC- and SEC-purified hemagglutinin trimers in phage PBS (100 mM NaCl, 32 mM Na2HPO4, 17 mM Na2HPO4, pH 7.2) were put into flat-bottom microtiter plates (ImmunoPlateMaxisorp, Nalgen Nunc International, Roskilde, Denmark) and incubated overnight at room temperature for coating. One hundred microliters of serum samples as serial dilutions starting at 1:100 was applied after blockage with 3% (*w*/*v*) bovine serum albumin (BSA), 0.05% (*v*/*v*) Tween20 in PBS (PBST) for 2 h and incubated at 25 °C for 1 h. Plates were washed 5 times with PBST, incubated with alkaline phosphatase-conjugated anti-chicken IgY diluted (35,000 times) in 1% (*w*/*v*) BSA and washed again. One hundred microliters of p-nitrophenyl phosphate (pNPP) substrate prepared in 0.1 M diethanolamine-HCl, pH 9.8 was applied. Plates were incubated at 37 °C for 1 h. The absorbance signal was measured at 405 nm. End-point titers were determined as the reciprocal highest serum dilutions that produced mean optical density values four-fold greater than the geometric mean of those from the negative control (injected with *N. benthamiana* WT plant extracts) sera.

### 2.10. Competitive ELISA

A commercial competitive ELISA (ID Screen^®^ Influenza H5 Antibody Competition) was used to detect anti-H5 chicken antibodies raised by the purified H5 trimers. The procedure followed the the manufacturer’s instructions (ID.vet).

### 2.11. Chicken Challenge Experiment with the Purified H5-TG Trimer

Seven chickens per group were intramuscularly immunized with Emulsigen^®^-D adjuvanted H5-TG trimer (10 µg antigen/chicken) in PBS at the second and fifth weeks of age. Seven chickens received neither antigens, adjuvants nor buffer (non-vaccinated group). At 7 weeks of age (two weeks after the second vaccination), each chicken was inoculated by 10^7^ plaque forming unit of HPAIV A/duck/Viet Nam/TG24-01/2005 (H5N1). All birds were clinically observed for 10 days. Clinical scoring was done as following: 0 for healthy birds, 1 for birds with one clinical sign (depression, respiratory disorders, diarrhoea, cyanosis of the comb or wattles, facial oedema or central nervous signs), 2 for birds that showed more than one clinical sign, and 3 for dead birds. The pathogenicity index (PI) was calculated as the sum of the daily arithmetic mean values divided by 10 (the number of observation days). The PI ranged from 0 (avirulent) to 3 (highly virulent).

The experiment was conducted in the biosafety level-3 animal facilities of the Friedrich-Löffler-Institut (FLI) following the German Regulations for Animal Welfare and authorized by the ethics committee of the State Office of Agriculture, Food Safety, and Fishery in Mecklenburg-Western Pomerania (permission number: 7221.3-1.1-051/12). The experiment was performed after approval from the authorities and the commissioner for animal welfare at the FLI representing the Institutional Animal Care and Use Committee (IACUC).

### 2.12. Chicken Challenge Experiment with Plant Crude Extracts Containing H5 Antigens

The hemagglutinin contents in plant extracts were semi-quantified by Western blotting. Plant extracts containing 70 ng of either H5HT oligomer-TP or H5HT trimer were selected for chicken immunization. In the control group, *N. benthamiana* WT plant extract that had the same amount of total soluble proteins as plant extracts containing H5HT oligomers were used. Plant extracts containing H5HT oligomer-TP or H5HT trimer, *N. benthamiana* WT plant extract, and commercial inactivated H5N1 virus vaccine (Navetco, Ho Chi Minh, Vietnam) were formulated with in-house water in oil adjuvant (NAVETCO) at 70% final concentration. To monitor the effect of the in-house water in oil adjuvant, plant extracts containing either H5HT oligomer-TP or H5HT trimer without adjuvant were included in the experiment. Twelve chickens (10 chickens for the negative control group) per group were intramuscularly immunized with antigens at the second and fifth weeks of age. Two weeks after the second vaccination, each chicken was inoculated by 10^6^ egg lethal dose 50 of HPAIV A/chicken/DL/NAVET 0292/2013 (H5N1). One day before viral inoculation, chicken sera were collected individually for hemagglutination inhibition and ELISA tests. All birds were observed for 10 days after the viral inoculation.

Experiments with chicken were conducted in accordance with the “3Rs” and the relevant rules of both Institute of Biotechnology (IBT, Ha Noi, Vietnam) and NAVETCO ethics committees under the decision number 07/2015/HĐ-NĐT. 

### 2.13. Statistical Analyses

Statistical analyses of HI data and ELISA results were performed using the Mann–Whitney rank-sum test (Sigma Plot software). *p*-values less than 0.05 were defined as significant.

## 3. Results

### 3.1. Trimerization Technology Keeps H5 Trimer and its Activity

Previously, we produced trimers of H5 hemagglutinin from the AIV strain A/Hatay/2004 (H5N1) in plants [16] by fusion of the trimeric GCN4-pII motif to the C-terminus of the H5 ectodomain. Here, this trimerization method was verified in another, more recent, H5 strain. We chose the sequence encoding the H5 ectodomain (aa 17–520) of A/duck/Vietnam/TG24-01/2005 (H5N1), codon optimized it for expression in *N. benthamiana*, and cloned it into a plant expression vector (Figure 1a and Tables S1 and S2). After insertion into a binary plasmid, agrobacterium-based transient H5 trimer expression in the leaf cells of *N. benthamiana* was induced by agro-infiltration. The H5 protein was purified by IMAC and analyzed by separation in SDS gels under reducing, denaturing conditions and Western blotting. A major band with an apparent molecular weight of 80 kDa and minor bands with molecular weights of 170 kDa and approximately 300 kDa, corresponding to a monomer, a dimer and a trimer of hemagglutinin, respectively, were detected (Figure 1b, -BS3 lane). The minor dimer and trimer bands could be explained by incomplete denaturation (Figure 1b, -BS3 lane).

The trimeric form of purified H5 proteins was determined by a crosslinking reaction using a BS3 (bissulfosuccinimidyl suberate) cross linker. H5 proteins were exposed to BS3 and crosslinked products were separated on a SDS gel under reducing and denaturing conditions, blotted and immunodetected using an anti-c-Myc monoclonal antibody. Immunoblot results reveal a single band with an apparent molecular weight of approximately 300 kDa corresponding to a trimer (Figure 1b, +BS3 lane). This result implies that the native structure of plant-derived H5 proteins is a H5 trimer.

The biological activity of the purified H5 trimers was assessed using hemagglutination assay. As shown in Figure 1c, purified H5 trimer together with inactivated H5N1 viruses (positive control) was able to agglutinate chicken red blood, while PBS (negative control) was not.

Taken together, the findings indicate that active and native H5 trimers of the A/duck/Vietnam/TG24-01/2005 (H5N1) strain can be obtained by the fusion of the trimeric GCN4-pII motif at the C-terminus.

### 3.2. Plant-Made Purified H5 Trimer Protects Chickens from HPAIV H5N1

Purified H5 trimers were tested for their ability to induce protective immunity against homologous highly pathogenic avian virus (HPAIV H5N1 Table S2) in challenge experiments with chickens. To address this, purified trimer and PBS (negative control group) were formulated with Emulsigen^®^-D adjuvant at 20% final concentration. Seven chickens per group were intramuscularly vaccinated with purified H5 trimers (10 µg antigen/chicken), and PBS at the second and fifth weeks of age. A group of seven non-vaccinated chickens were included. At one day old (before the first immunization) and seven weeks old (two weeks post second vaccination, and before the viral challenge), chicken sera were collected. Serum samples were examined using a commercial competitive ELISA against anti-H5 avian influenza antibodies (ID Screen^®^ Influenza H5 Antibody Competition, ID.vet). As summarized in Table 1, no anti-H5 antibodies were detected in serum samples at day one of age in all groups. As expected, anti-H5 antibodies were not detected in sera from the non-vaccinated group and from the negative control group (immunization with PBS) at seven weeks of age (before challenge). However, all sera collected immediately before virus challenge from chicken immunized with purified H5 trimers showed seroconversion detected by a commercial competitive ELISA. Chickens immunized with purified H5 trimers induced H5 specific antibodies, while control animals did not (Table 1).

**Figure 2:**
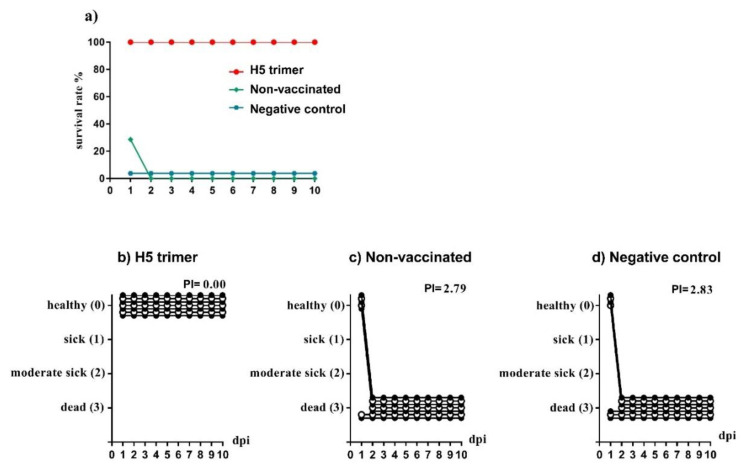
Purified H5-TG trimer protects chickens from HPAIV H5N1. Chickens immunized either with purified H5 trimer, or PBS, and non-vaccinated chicken were challenged with 10^7^ plaque forming units of HPAIV A/duck/Vietnam/TG24-01/2005 (H5N1). Chicken survival rate was observed for 10 days after the viral challenges (**a**). Daily clinical scoring was recorded as following: 0 for healthy birds, 1 for birds with one clinical sign (depression, respiratory disorders, diarrhea, cyanosis of the comb or wattles, facial oedema or central nervous signs), 2 for birds that showed more than one clinical sign, and 3 for dead birds. The average scores of all animals per group and day post virus infection are indicated. The pathogenicity index (PI) was calculated as the sum of the daily arithmetic mean values divided by 10 (the number of observation days). (**b**) The PI of chickens immunized with purified H5 trimer, (**c**) The PI of non-vaccinated chickens, (**d**) The PI of chickens immunized with PBS. Dpi: day post viral inoculation.

Two weeks after the second vaccination, each chicken was inoculated by 10^7^ plaque forming units of HPAIV H5N1 (Table S2). All birds were observed for 10 days for clinical signs. As shown in Figure 2a, all chickens in the PBS and untreated groups died within two days after viral challenge. Pathogenicity indexes of these two groups were 2.79, and 2.83, respectively (Figure 2c,d). All chickens that were immunized with H5 trimer were fully protected against the lethal challenge. The PI of this group was zero (Figure 2b). Thus, the purified trimer antigen was immunogenic to protect all vaccinated chickens from morbidity and mortality following a severe HPAIV H5N1 challenge.

### 3.3. Multimerization of H5-TG Trimer in Plants

It has been demonstrated that the immunogenicity of influenza hemagglutinins depends on their oligomeric nature. High molecular weight hemagglutinins (oligomers) induced stronger antibody responses than trimers and monomers [12,13]. In addition, oligomeric levels of influenza hemagglutinin depended on production cell sources [12], or on specific hemagglutinin fragments used [13]. Until now, there are no generic methods that can produce influenza hemagglutinin oligomers.

**Figure 3:**
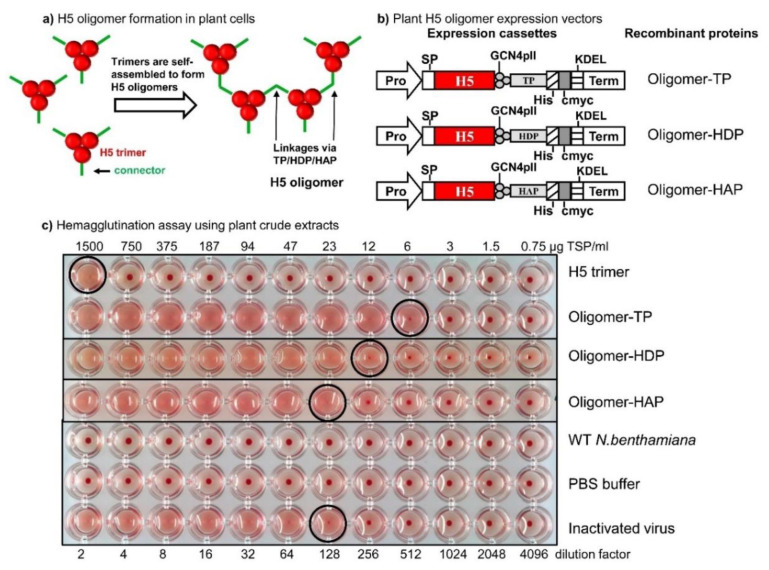
Production and characterization of H5 trimers and H5 oligomers from plants. (**a**) Model of H5 oligomer formation by H5 trimers self-assembled. H5 trimers are connected by cysteine residues (via disulfide bonds) in the tail piece sequence (TP) from the C-terminal sequence of the mouse IgM [18] (Tables S1 and S2), homoantiparallel peptides (HAP) [19,20,21] (Tables S1 and S2), and homodimer proteins (HDP) [22,23] (Tables S1 and S2). (**b**) Expression cassettes for H5 oligomers. Starting with the construct mentioned above, the tail piece (TP) sequence of the mouse IgM antibody [18], the homoantiparallel peptide (HAP) sequence [19,20,21], or the homodimer proteins (HDPs) sequence [22,23] were introduced directly after the GCN4pII trimer motif. The legumin B4 signal peptide (SP) and the KDEL motif caused ER retention. Pro: cauliflower mosaic virus 35S ubiquitous promoter; term: cauliflower mosaic virus 35S terminator. (**c**) Screening for H5 oligomers by the hemagglutination assay. Two-fold serial dilutions of plant extracts that contained H5 trimers or H5 oligomers, WT, PBS buffer and inactivated virus were mixed with the given amounts of chicken red blood cells, and hemagglutination was recorded. Circles indicate hemagglutination titers. Inactivated rg A/swan/Germany/R65/2006 (H5N1) virus, WT: wild-type *N. benthamiana*.

We hypothesized that stabilized H5 trimers can be linked via connectors that are introduced after the GCN4pII trimeric motif to form H5 oligomers, outlined in Figure 3a,b. The connectors are disulfide bonds formed by cysteine residues in the tail piece sequence (TP) from the C-terminal sequence of IgM [18] (Figure 3b and Tables S1 and S2), homoantiparallel peptides (HAP) [19,20,21] (Figure 3b and Tables S1 and S2), and homodimer proteins (HDP) [22,23] (Figure 3b and Tables S1 and S2).

After transient expression of the relevant constructs (Figure 3b) in *N. benthamiana* leaves, the expression of all four proteins was confirmed. Expression levels of these proteins (H5 trimer, oligomer-TP, oligomer-HDP, and oligomer-HAP) were approximately 0.02, 0.02, 0.003, and 0.003% of totally soluble, respectively, as semi-quantitatively estimated by Western blot (Figure S1). The formation of hemagglutinin oligomers was analyzed in plant crude extracts via hemagglutination assay. In the hemagglutinin oligomers, cross-links between hemagglutinin trimers were formed via disulfide bonds, homoantiparallel peptides or homodimer proteins under non-reducing conditions in the ER, while influenza trimers lacked these linkages. Crude extracts from wild-type *N. benthamiana* and PBS buffer (negative controls) did not show hemagglutination activity (Figure 3c), while the hemagglutination (HA) titer of the H5 trimer plant extract was as low as two. However, the HA titers of the oligomer-TP, oligomer-HDP, and oligomer-HAP plant extracts dramatically increased to 512, 128, and 256, respectively.

**Figure 4:**
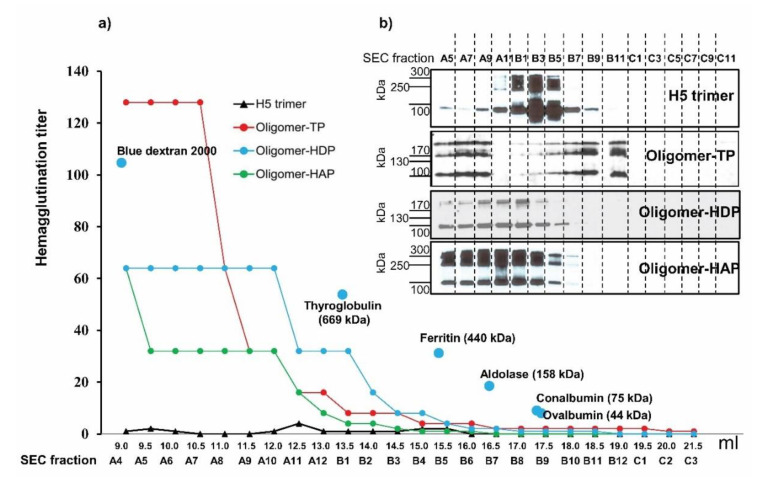
Characterization of H5 trimer and H5 oligomers by size exclusion chromatography. IMAC-purified H5 trimers and three H5 oligomers were separated on Superose™ 6 increase 10/300GL according their sizes. The standard proteins loaded on the column to estimate the native molecular weights of target proteins are presented by light blue dots. SEC fractions of H5 trimer, H5 oligomer-TP, oligomer-HDP, and oligomer-HAP were analyzed by hemagglutination assay (**a**) and Western blot (**b**). The hemagglutination titer of each single fraction (from A4 to C3) of H5 trimer, H5 oligomer-TP, oligomer-HDP, and oligomer-HAP are given. Presence of hemagglutinin proteins in the SEC fractions was confirmed by Western blotting by use of an anti-c-Myc monoclonal antibody.

The potential oligomers of the H5 trimers generated by three strategies (oligomer-TP, oligomer-HDP, and oligomer-HAP) were further characterized under native conditions. After affinity purification, all three potential oligomers were separated by SEC, and the fractions were characterized by Western blotting and hemagglutination titer estimation. As demonstrated in Figure 4, high hemagglutination titers were observed in early fractions A4 to A10 of all three H5 oligomers. These fractions reflect high molecular weight of recombinant proteins. Notably, the highest molecular weight (fraction A4) corresponds to one of the highest hemagglutination titer values (Figure 4). SEC fractions of H5 trimer did not show high molecular weights or high hemagglutination titers in the corresponding fractions (A4 to A10) of H5 oligomers (Figure 4). The fractions of oligomer-TP, oligomer-HDP, and oligomer-HAP were analyzed by SDS-PAGE and in parallel with fractions of H5 trimer by Western blot. Very high molecular weight hemagglutinins were present in fractions A9 to A5 (>700 kDa) of all three H5 oligomers, while no high molecular weight proteins were detected after H5 trimer analysis. Based on the size of trimeric H5 (~400 kDa) determined by SEC, and the size of H5 oligomers determined by SEC, the number of H5 trimers integrated in H5 oligomers via disulfide bonds, homodimer protein, and homoantiparallel peptides ranged from one to four, indicating that the H5 oligomers were a mixture in respect to the molecular weight.

### 3.4. High Immunogenicity of H5 Oligomers in Plant Crude Extracts in Mice

**Figure 5:**
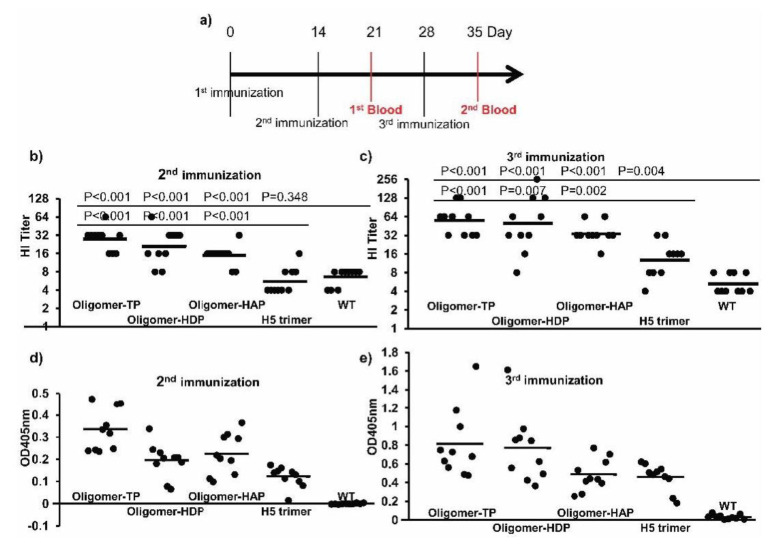
Immune responses of mice immunized with plant crude extracts containing H5 trimers and H5 oligomers. (**a**) Mouse immunization and bleeding schedule. Each mouse was immunized either with crude extracts containing 0.1 µg (oligomer-TP, oligomer-HDP, oligomer-HAP or H5 trimer, respectively) formulated with the Emulsigen^®^-D adjuvant at 20% final concentration. In the negative control group, mice were injected with *N. benthamiana* wild-type leaf extracts formulated with the Emulsigen^®^-D adjuvant at 20% final concentration. Mice were vaccinated with the formulated vaccines at days 0 and boosted at days 14 and 28. Sera were collected one week after the second and third immunizations. (**b**,**c**) Antibody responses estimated by hemagglutination inhibition assay after the second and third immunization. Measurement of neutralizing antibodies raised by injection of different extracts into mice after two (**b**) and three (**c**) immunizations by hemagglutination inhibition assay. Single dots represent the HI titers of single serum samples and bars are the geometric mean titers of each test group. (**d**,**e**) Antibody responses estimated by indirect ELISA after the second and third immunization. Measurement of antibodies raised by injection of different extracts into mice after two (**d**) and three (**e**) immunizations by indirect ELISAs. Single dots represent the OD405nm value of single serum samples and bars are the average OD405nm values of each test group.

As represented in Figure 4, hemagglutination titers of hemagglutinin oligomers in plant extracts were high in comparison with those of inactivated viruses. We intended to minimize down-stream processing to suit the economical demands for animal vaccine development. Crude extracts containing trimeric and oligomeric hemagglutinins were tested in vaccination and challenge experiments. To address this, mouse immunization experiments were conducted to test immunogenicity of all four candidate vaccines: H5 oligomer-TP, oligomer-HDP, oligomer-HAP, and H5 trimer in plant lysates in terms of immune responses. Groups of 12 C57Bl6/J mice each were immunized three times with either wild-type plant crude extracts or crude extracts from plants expressing H5 oligomer-TP, oligomer-HDP, oligomer-HAP, and H5 trimer, respectively (Figure 5a). Mouse sera were collected after the second and third immunization for ELISA and hemagglutination inhibition tests. The data we achieved suggested which H5 candidate vaccine should go further for virus challenge experiments.

Neutralizing antibodies (inhibiting hemagglutination) raised by oligomer-TP, oligomer-HDP, oligomer-HAP, H5 trimer, and WT crude extracts were then measured. Due to unavailability of the A/duck/Viet Nam/TG24-01/2005 (H5N1) inactivated virus, the heterologous inactivated virus strain rg A/swan/Germany/R65/2006(H5N1) was used for the HI assay. The two strains shared 96% deduced hemagglutinin amino acid sequence similarity. As shown in Figure 5b, neutralizing antibodies inhibiting hemagglutination were produced in mice immunized with H5 oligomer-TP, oligomer-HDP, and oligomer-HAP crude extracts after the second immunization. HI geometric mean serum antibody titers (HI GMTs) of mice immunized with H5 oligomer-TP, oligomer-HDP or oligomer-HAP crude extracts were 27.9, 21.1, and 15.0, respectively (Figure 5b). HI GMTs of mice immunized with H5 trimer and WT crude extracts were as low as 5.7 and 6.5, respectively. These HI GMTs were significantly lower than those of mice vaccinated with oligomer-TP, oligomer-HDP or oligomer-HAP crude extracts. Following the third immunization, the HI GMTs of oligomer-TP, oligomer-HDP, and oligomer-HAP groups were higher than after the second immunization, especially in the sera derived from mice vaccinated with oligomer-TP, oligomer-HDP or oligomer-HAP crude extracts compared to sera from mice immunized with trimer crude extracts or WT crude extracts (Figure 5c). The HI GMTs of these mice were 56.0, 48.5 and 34.3, while the negative control sera and H5 trimer HI titers were as low as 5.3 and 13, respectively. Again, neutralizing antibody titers induced by the H5 oligomer crude extracts were significantly higher than those induced by H5 trimer crude extracts (Figure 5c). A four-fold increase in HI GMTs was observed in sera from mice vaccinated with oligomer-TP, oligomer-HDP or oligomer-HAP crude extracts compared to sera from mice immunized with WT crude extracts. A four-fold increase in HI titers is associated with two-fold decrease in the risk of infection [27] and defined as seroconversion [28].

The humoral immune responses were tested against purified SEC hemagglutinin H5 by an indirect ELISA (Figure 5d,e). No immune responses against hemagglutinin H5 were, as expected, detected after immunization with the WT crude extract, whereas specific binding to purified hemagglutinin H5 was measured after immunization with oligomer-TP, oligomer-HDP or oligomer-HAP or H5 trimer crude extracts (Figure 5d). Immune responses increased after the third immunization (Figure 5e).

Taken together, plant crude extracts containing H5 oligomers and trimers induced specific antibodies against H5. Oligomers induced neutralizing antibodies with titers significantly higher compared to trimers. Here, oligomer-TP performed best.

### 3.5. High Immunogenicity of H5 Oligomers in Plant Crude Extracts in Chickens

**Figure 6:**
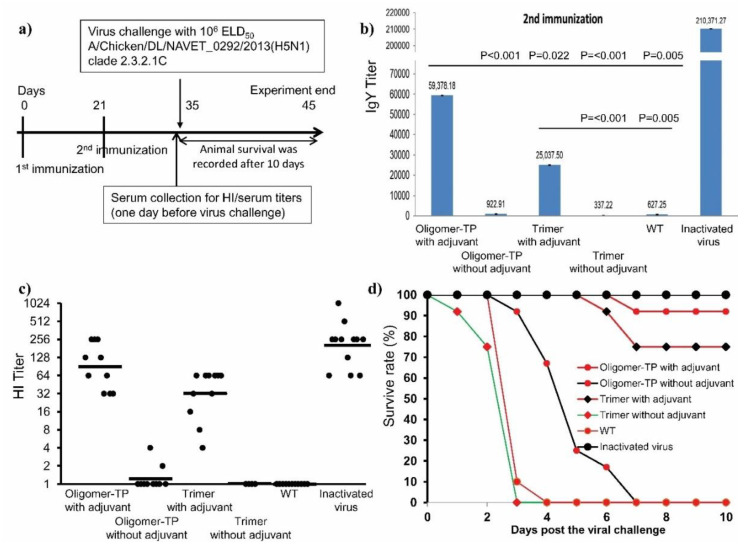
Immune responses of chickens immunized with plant crude extracts containing H5 trimers and H5 oligomers. (**a**) Chicken immunization scheme. Twelve chickens per group were intramuscularly immunized with adjuvant and non-adjuvant H5 oligomer-TP and H5 trimer plant crude extracts at days 0 and 21. The adjuvant WT plant crude extract (negative control) and inactivated virus (positive control) were included. Thirteen days after the second immunization, all chicken sera were collected for immune response analyses. Fourteen days after the second immunization, all chickens were challenged with HPAIV A/Chicken/DL/NAVET_0292/2013 (H5N1) at a 10^6^ ELD50 dose. The chicken survival rate was recorded 10 days after viral challenge. Chicken sera collected after the second immunization and before virus challenge were analyzed on the basis of ELISA measuring the specific IgY titer (**b**), HI titer estimation (**c**) and the chicken survival rate (**d**).

During this study, a new H5N1 strain of clade 2.3.2.1 was introduced into Vietnam from Southern China. The H5 sequence (H5HT) of the A/duck/Viet Nam/HT2/2014 (H5N1) strain (clade 2.3.2.1c) [29] (Table S2) was therefore selected, synthesized, and cloned into plant expression vectors to produce H5HT trimer and H5HT oligomer-TP vaccines that are specific for this newly circulating strain in Vietnam. The expression of the H5HT trimer and H5HT oligomer-TP was confirmed by Western blotting. The HA titers of both the H5HT oligomer-TP crude extracts were again higher than those of H5HT (data not shown). The H5 contents of the crude extracts were then semi-quantified by Western blotting, and the plant crude extracts containing H5HT trimers or H5HT oligomer-TP as well as the WT plant crude extract (negative control) and the commercial inactivated H5N1 virus vaccine (positive control) were formulated with an in-house water in oil adjuvant (NAVETCO). Plant crude extracts containing H5HT trimers and H5HT oligomer-TP without an adjuvant were also included to analyze the effect of the adjuvant. The resulting emulsions were used to immunize chickens twice (Figure 6a). The chicken immune responses against purified hemagglutinin H5 after the second immunization were detected by Western blotting (Figure S2) and measured by an indirect ELISA. As shown in Figure S2, H5-specific antibodies raised by the adjuvant H5HT oligomer-TP and H5HT trimer crude extracts and inactivated virus have been detected by Western blotting, whereas no H5 specific bands were visible in case of sera from chickens administrated by non-adjuvant H5HT oligomer-TP and H5HT trimer crude extracts and WT crude extract (Figure S2). This result indicates that the adjuvant H5HT oligomer-TP and H5HT trimer crude extracts induced strong H5-specific antibodies in chickens.

To confirm these observations, H5-specific chicken IgY titers of all chicken groups were estimated by an indirect ELISA. In Figure 6b, the chicken IgY responses elicited by the non-adjuvant H5HT oligomer-TP, H5HT trimer crude extracts and WT plant crude extracts were poor. Their endpoint IgY titers were 922.91, 337.22, and 727.25, respectively. In contrast, the administration of the adjuvant H5HT oligomer-TP, H5HT trimer crude extracts and the commercial inactivated H5N1 virus vaccine elicited high IgY responses. Their IgY titers reached to 59 378.18, 25 037.50, and 210 371.27, respectively. IgY immune responses elicited by the adjuvant H5HT oligomer-TP, H5HT trimer crude extracts were significant with those triggered by non-adjuvant H5HT oligomer-TP, H5HT trimer crude extracts. Notably, IgY immune responses induced by the adjuvant H5HT oligomer-TP crude extracts were significantly better than those elicited by the adjuvant H5HT trimer crude extracts (Figure 6b).

Neutralizing antibodies were measured by the hemagglutination inhibition test. Inactivated A/Chicken/DL/NAVET_0292/2013 (H5N1) virus was used in the HI test. A/duck/Viet Nam/HT2/20144 (H5N1) and A/Chicken/DL/NAVET_0292/2013 (H5N1) are both grouped in clade 2.3.2.1c and share 98% similarity of the deduced hemagglutinin amino acid sequence (Table S2). The data presented in Figure 6c confirm the indirect ELISA results. Both the adjuvant H5HT oligomer-TP and H5HT trimer crude extracts induced neutralizing antibodies against the inactivated virus in chickens. HI GMTs of these groups were 90.5, and 32.0, respectively. HI GMTs of chickens immunized with the non-adjuvant H5HT oligomer-TP and H5HT trimer crude extracts were as low as 1.2 and 1.0, respectively (Figure 6c). These titers were similar to those of chickens vaccinated with the WT crude extract and much lower than those of chickens immunized with the adjuvant H5HT oligomer-TP and H5HT trimer crude extracts and the positive control group (HI GMT = 203.2) (Figure 6c). These results indicate that both the adjuvant H5HT oligomer-TP and H5HT trimer crude extracts induced neutralizing antibodies in chicken.

**Figure 7:**
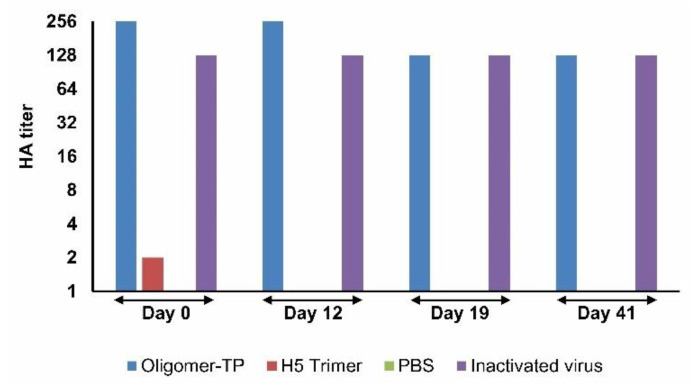
Stability analysis of H5 oligomers and H5 trimer crude extracts. Plant crude extracts containing either oligomer-TP or trimers were kept on ice and stored at 4 °C for 0, 12, 19, and 41 days and the hemagglutination titers were estimated. PBS and inactivated virus were included in hemagglutination tests at all time points as negative and positive controls, respectively.

Two weeks after the second immunization, all birds were challenged with 10^6^ egg lethal dose 50 (10^6^ ELD50). All negative control animals died within four days after viral challenge. Animals that were vaccinated with the non-adjuvant H5HT trimer crude extract died within three days. Animals vaccinated with the non-adjuvant H5HT oligomer-TP crude extract also died, but with a delay of 1–3 days. In contrast, animals vaccinated with adjuvant H5HT trimer and the H5HT oligomer-TP crude extracts showed high survival rates. Nine out of twelve animals (75%) immunized with adjuvant H5HT trimer crude extracts and eleven out of twelve animals (92%) immunized with adjuvant H5HT oligomer-TP crude extracts were protected from lethal disease. One animal died with a delay at day six after virus challenge. All chickens in the positive control group (immunized with the standard vaccine) survived. To monitor the stability of H5 antigens in plant extracts, two independent experiments were performed (Figure 7 and Figure S3). H5HT oligomer-TP and H5HT trimer-containing extracts were kept on ice and stored at 4 °C. The hemagglutination titers of the plant lysate containing H5HT oligomers were observed after 41 days (Figure 7). The titer of the oligomer extract was slightly changed from 256 (day 0) to 128 (day 19), but was stable until day 41. The stability was confirmed in a second experiment (Figure S3).

## 4. Discussion

Many efforts have been made to explore multivalent antigens (oligomers) in clinical vaccine development. Multivalent antigen presentation can be achieved by using nanoparticles for antigen display [30] or by attachment to biodegradable polymer materials [31]. Generally, multivalent antigen platforms have generated better immune responses due to their superantigen presentation [30,32]. We generated multivalent antigens in plants using different methods. All of the H5 oligomers were derived from H5 trimers, which were connected by disulfide bonds, homodimer protein, and homoantiparallel peptides to form multivalent antigens. Unlike oligomeric formation based on Strep-Tactin^®^XT-Streptag^®^II interaction [15], which requires purified antigens and is covered by patents [33,34] that lead to high costs for veterinary vaccines, our oligomeric generation methods were self-assembling in plant cells, and an open technology. This could encourage veterinary vaccine manufacturers to use this technology for veterinary vaccination in the future.

Recombinant H5 expression in eukaryotic expression systems results in superior antigens compared to other expression systems in terms of posttranslational modifications such as disulfide bond formation, glycosylation, protein folding and trimerization. We and others have shown that recombinant influenza H5 was N-glycosylated both in plant cells [35,36] and animal cells [37]. Plant N-linked glycan profiles of recombinant H5 depend on storage compartments. The recombinant ER retention H5 proteins carried the predominant oligo-mannose type glycans (70–72%), and 27–30% the plant-specific complex type glycans with core α(1,3)-fucose, core β(1,2)-xylose. H5 virus-like particle (stored in the apoplast compartment) had complex type glycans with core α(1,3)-fucose, core β(1,2)-xylose and Lewis -acid-catalyzed glycosylations. However, neither hypersensibility nor IgG, or IgE induction against plant glycan epitopes was observed [38]. In animal cells, HEK293T cell-produced recombinant H5 carried complex glycans that often terminally combine sialic acids [37]. Interestingly, de Vries and colleagues reported that recombinant H5 proteins bearing mannose glycans induced significantly lower HI titers than H5 proteins carrying complex glycans or single N-acetylglucosamine side chains. Here we showed that H5 oligomers had better performance in terms of neutralizing antibody induction than H5 trimers. However, N glycan profiles of both H5 trimers and H5 oligomers were not analyzed. Therefore, their glycosylation states should be addressed to monitor differences in the future. The N-glycan differences in H5 oligomers and H5 trimers could play a role in their immunogenicity.

The results presented in the actual study and data provided by others [39] showed that designed highly immunogenic antigens produced at high level *in planta* allow the direct use of plant extracts containing antigens for animal vaccination. However, *N. benthamiana* plants are known to contain alkaloids, especially a high amount of nicotine (90% of total alkaloids), and anabasine (8.4%) [40]. Nicotine content is varying at different experimental conditions such as plant growth, extraction methods or *N. benthamiana* variants. Although all animals in our study were healthy and alive after vaccination with plant extracts, nicotine contents present in plant extracts should be monitored in the future studies dependent on the extraction method to further improve this vaccination strategy.

## 5. Conclusions

Our results clearly show the potential of H5 oligomers in plant crude extracts for the development of cheap protective veterinary vaccines meeting the demands of highly effective vaccines, simple and severe production strategies and efficient and applicable methods for distribution and use. Together with the high stability of the H5 oligomers in plant crude extracts, this is a very well-founded concept, especially for developing countries. The developments and experiments in Vietnam strengthen the value of these data in the fight against avian flu as an international goal. Such a vaccine against avian flu has the potential to decrease virus shedding in poultry significantly and thus could help to build herd immunity. As a zoonosis, avian flu is of significant importance for human medicine, especially because of the rather high mortality rate of infected patients. In the light of specific epidemiologic conditions in south-east Asia, such a vaccine could be very useful in reducing the danger of infection in human beings.

## Figures and Tables

**Table 1:** Immunological analyses of birds vaccinated with purified H5 trimers by a commercial competitive ELISA. Chicken sera were immediately collected before the viral challenge. * Some sera were fatty and could not be examined by a commercial competitive ELISA.

Group	Seroconversion (One Day Old)	Seroconversion * (Before the Viral Challenge)
Purified H5 trimer	0/7	5/5
Non vaccinated	0/7	0/5
PBS	0/7	0/5

## Supplementary Materials

The following are available online at https://www.mdpi.com/2076-393X/8/4/593/s1. Figure S1: Expression of recombinant influenza H5 variants in plants, as demonstrated by Western blot, Figure S2: Specific immune responses induced by H5 trimers and oligomers in plant crude extracts in chicken, Figure S3: Stability analysis of crude extracts. Plant crude extracts containing either Oligomer-TP, Oligomer-HDP, Oligomer-HAP or trimers have been stored at 4 °C for 0, 12 and 26 days and the hemagglutination titers have been estimated, Table S1: Plant expression constructs and their features, Table S2: H5 amino acid sequences of different used H5N1 strains and recombinant H5 proteins inclusive functional motives.
