# Supplementary material for: Immunization with Plant-Derived Multimeric H5 Hemagglutinins Protect Chicken against Highly Pathogenic Avian Influenza Virus H5N1
Source: Vaccines (Basel). 2020 Oct 9;8(4):593. doi: 10.3390/vaccines8040593 (PMC7712794; doi:10.3390/vaccines8040593)
Supplement: Supplementary file 1 [file vaccines-08-00593-s001.pdf]

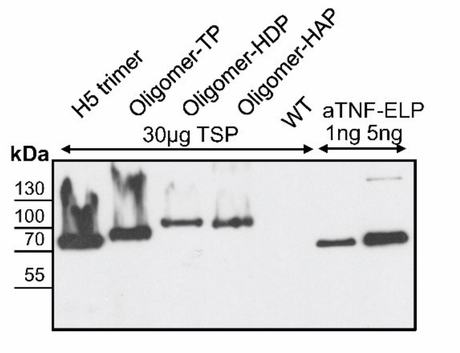

Figure S1. Expression of recombinant influenza H5 variants in plants, as demonstrated by Western blot. Extracted proteins from leaf materials expressing H5 trimer, oligomer-TP, oligomer-HDP, oligomer-HAP and *N. benthamiana* (a negative control) were separated in a reduced SDS-PAGE gel. The known amounts of purified aTNF-ELP [25] was included as a positive control and semi-quantification. Recombinant proteins were visualized by Western blotting with an anti-c-Myc monoclonal antibody.

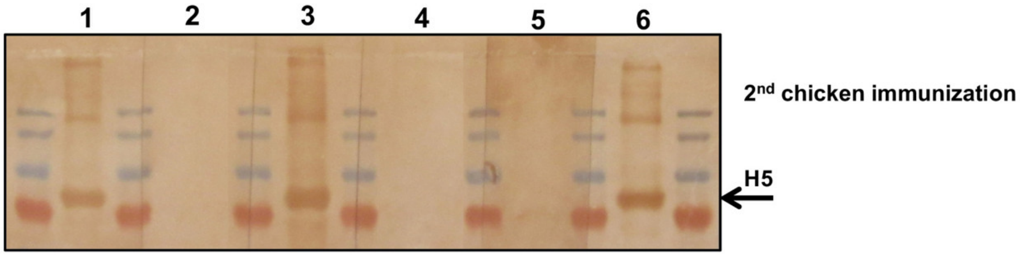

Figure S2. Specific immune responses induced by H5 trimers and oligomers in plant crude extracts in chicken. H5-specific binding of antibodies from mixtures of 12 sera raised against corresponding plant extracts as demonstrated by Western blot. To detect the HA-specific IgG mouse antibody raised by different plant extract variants and commercial NAVET-VIFLUVAC (Navetco, Ho Chi Minh, Vietnam), 700 ng of the SEC-purified H5TG proteins were separated on 6 lanes of a reducing SDS–PAGE gel (10% polyacrylamide) and transferred to a nitrocellulose membrane. The membrane was blocked with a 5% (w/v) fat-free milk powder dissolved in a PBS buffer for 2 h. Then, each lane of the membrane was isolated by cutting and incubated at room temperature for 90 min with a 1:20 dilution of a mixture of sera from twelve chickens of each group after the second immunizations. The membranes were incubated with a 1:5000 dilution of anti-chicken IgY (whole molecule) alkaline phosphatase secondary antibody for 1 h at room temperature. Specific signals were detected by incubating membranes with 3,3–diaminobenzidine (DAB, Thermo Scientific Pierce) dissolved in 0.05 M Tris–HCl and 0.04% hydrogen peroxide for 10 min in the dark.1: Chicken sera raised by plant extract containing H5HT oligomer-TP and adjuvant. 2: Chicken sera raised by plant extract containing H5HT oligomer-TP. 3: Chicken sera raised by plant extract containing H5HT trimer and adjuvant. 4: Chicken sera raised by plant extract containing H5 trimer. 5: Sera raised by WT plant extract and adjuvant. 6: Sera raised by NAVET-VIFLUVAC and adjuvant.

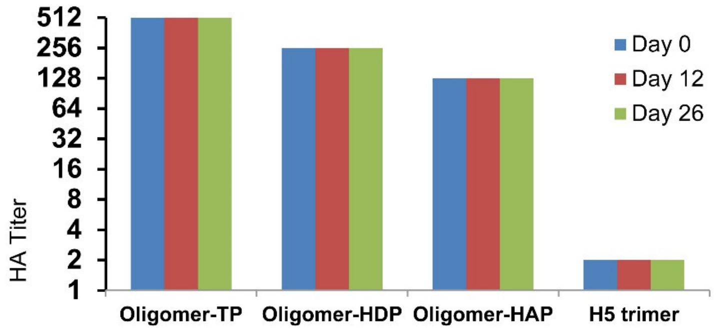

Figure S3. Stability analysis of crude extracts. Plant crude extracts containing either oligomer-TP, oligomer-HDP, oligomer-HAP or trimers have been stored at 4°C for 0, 12 and 26 days and the hemagglutination titers have been estimated.

Table S1. Plant expression constructs and their features.

	Connector	Features of connector	Expression confirmed by Western blot	Hemagglutination titers from plant crude extracts
H5 trimers				
01	no	H5 was trimerized by GCN4-pII motif to form H5 trimers.H5 sequence was from A/duck/Viet Nam/TG24-01/2005(H5N1) strain.	+	2
02	no	H5HT was trimerized by GCN4-pII motif to form H5TH trimers. H5HT was from A/duck/Viet Nam/HT2/2014(H5N1) strain	+	2
H5 trimers are connected by disulfide bonds to form H5 oligomers				
01	Mouse IgM Fc domain	Cμ3-Cμ4tp (Ile393-Tyr576) of the mouse IgM heavy chain. This domain contains interchain disulfide bridges at Cμ3C414 and Cμ4tpC575. The Cμ3-Cμ4tp (Ile393-Tyr576) domain [18] was c-terminally fused to trimerized H5. The interchain disulfide bridge from Cμ3C414 and C575TP led to form H5 oligomer.	+	64
02	TP (tail piece) sequence of the mouse IgM heavy chain	TP domain of the mouse IgM heavy chain consists of 19 amino acids. It contains C575 expecting to form the interchain disulfide bridge [18]. A TP domain was c-terminally fused to trimerized H5. The interchain disulfide bridge from C575TP led to form H5 oligomer-TP	+	512
H5 trimers are connected by homodimer proteins/ homodimer peptides				
04	Hoefavidin	Hoefavidin is one member of the dimeric avidin subfamily [23]. Hoefavidin was c-terminally fused	+	256

05		to trimerized H5. Dimeric formation of hoefavidin-trimerized H5 led to build H5 oligomer-HDP.		32
	Shwanavidin	Shwanavidin is another member of the dimeric avidin subfamily [22]. Shwanavidin was c-terminally fused to trimerized H5. Dimeric formation of shwanavidin - trimerized H5 led to build H5 oligomers.	+	
	GCN4			64
H5 trimers are connected by homoantiparallel peptides				
06	4OH9	The human Mst2 Sarah homoantiparallel dimer (PDB ID: 4OH9) contains 39 amino acids [21]. The motif was c-terminally fused to trimerized H5.	ND	Negative
07	1XSX	Homoantiparallel dimer motif from a hyperthermophile coiled-coil DNA-binding protein Sso10a (PDB ID: 1XSX) contains 31 amino acids [19]. The motif was c-terminally fused to trimerized H5 to form H5 oligomer-HAP	ND	128
08	2LW9	Homoantiparallel dimer motif from myosin X (PDB ID: 2LW9) contains 44 amino acids [20]. The motif was c-terminally fused to trimerized H5.	ND	16

Table S2. H5 amino acid sequences of different used H5N1 strains and recombinant H5 proteins inclusive functional motives.

>A/duck/Viet Nam/TG24-01/2005(H5N1), designated as H5TG
DQICIGYHANNSTEQVDTIMEKNVTVTHAQDILEKTHNGKLCDLDGVKPLILRDCSVAGWLLGNPMCDEFINV
PEWSYIVEKANPVNDLCYPGDFNDYEELKHLLSRINHFEKIQIIPKSSWPSHEASLGVSSACPYQGKSSFFRNVVW
LIKKNSTYPTIKRSYNNTNQEDLLVLWGIHHPNDAAEQTKLYQNPTTYISVGTSTLNQRLVPRIATRSKVNGQSG
RMEFFWTILKPNDAINFESNGNFIAPEYAYKIVKKGDSTIMKSELEYGNCNTKCQTPMGAINSSMPFHNIHPLTI
GECPKYVKSNRLVLATGLRNSPQRERRGLFGAIAGFIEGGWQGMVDGWYGYHHSNEQGSGYAADKESTQKAI
DGVTNKVNSIIDKMNTQFEAVGREFNNLERRIENLNKKMEDGFLDVWTYNAELLVLMENERTLDFHDSNVKN
LYDKVRLQLRDNAKELGNGCFEFYHKCDNECMESVRNGTYDYPQYSEEAKLKREEISGVKLEGS

>A/duck/Viet Nam/HT2/2014(H5N1)
DHICIGYHANNSTEQVDTIMEKNVTVTHAQDILEKTHNGKLCDLNGVKPLILKDCSVAGWLLGNPLCDEFINV
PEWSYIVEKPNPANDLCYPGNFNDYEELKHLLSRINHFEKIQIIPKDSWSNHEASLGVSAACSYQGNSSFFRNVV
WLIKKDNAYPTIKKGYNNTNREDLLILWGIHHPNDEAEQTRLYQNPTTYISIGTSTLNQRLVPKIATRSKINGQS
GRIDFFWTILKPNDAIHFESNGNFIAPEYAYKIVKKGDSTIMRSEVEYGNCNTRCQTPIGAINSSMPFHNIHPLTIG
ECPKYVKSNKLVLATGLRNSPQRERRRKRGLFGAIAGFIEGGWQGMVDGWYGYHHSNEQGSGYAADKESTQK
AIDGVTNKVNSIIDKMNTQFEAVGREFNNLERRIENLNKKMEDGFLDVWTYNAELLVLMENERTLDFHDSNV
KNLYDKVRLQLKDNAKELGNGCFEFYHKCNNECMESVRNGTYDYPQYSEEARLKREEISGVKLESI

>rg A/swan/Germany/R65/ 2006(H5N1), designated as H5R65. This is the reverse genetics strain. Inactivated form viruses of this strain were used to measure mouse HI titers.
MEKIVLLFAIVSLVKSDQICIGYHANNSTEQVDTIMEKNVTVTHAQDILEKTHNGKLCDLDGVKPLILRDCSVA
GWLLGNPMCDEFLNVPEWSYIVEKINPANDLCYPGNFNDYEELKHLLSRINHFEKIQIIPKSSWSDHEASSGVSS
ACPYQGRSSFFRNVVWLIKKDNAYPTIKRSYNNTNQEDLLVLWGIHHPNDAAEQTRLYQNPTTYISVGTSTLN

QRLVPKIATRSKVNGQSGRMEFFWTILKPNDAINFESNGNFIAPENAYKIVKKGDSTIMKSELEYGNCNTKCQTP
IGAINSSMPFHNIHPLTIGECPKYVKSNRLVLATGLRNSPQGERRRKKRGLFGAIAGFIEGGWQGMVDGWYGY
HHSNEQGSGYAADKESTQKAIDGVTNKVNSIINKMNTQFEAVGREFNNLERRIENLNKKMEDGFLDVWTYNA
ELLVLMENERTLDFHDSNVKNLYDKVRLQLRDNAKELGNGCFEFYHRCDNECMESVRNGTYDYPQYSEEARL
KREEISGVKLESIGTYQILSIYSTVASSLALAIMVAGLSLWMCSNGSLQCRICI

>A/chicken/DL/NAVET 0292/2013(H5N1), designated as H5NAVET

This is the wild type, highly pathogenic avian influenza virus H5N1. The strain was used to challenge plant crude extract vaccinated chickens.

MEKIVLLFATISLVKSDHICIGYHANNSTEQVDTIKEKNVTVTHAQDILEKTRNGKLCDLNGVRPLILKDCRVSG
WLLGNPLCDEFSIVPEWSYIVEKANPANDLCYPGNFNDYEELKHLLSRINHFEKIQIITKDSWSDHEASLGVSAA
CSYQGNSSFFRNVVWLIKKDNAYPTIKKGYNNTNREDLLILWGIHHPNDEAEQTRLYQNPTTYISIGTSTLNQRL
VPKIATRSKINGQSGRIDFFWTILKPNDAIHFESNGNFIAPEYAYKIVKKGDSTIMRSEVEYGNCNTRCQTPIGAIN
SSMPFHNIHPLTIGECPKYVKSNKLVLATGLRNSPQRERRRKRGLFGAIAGFIEGGWQGMVDGWYGYHHSNEQ
GSGYAADKESTQKAIDGVTNKVNSIIDKMNTQFEAVGREFNNLERRIENLNKKMEDGFLDVWTYNAELLVLM
ENERTLDFHDSNVKNLYDKVRLQLKDNAKELGNGCFEFYHKCNNECMESVRNGTYDYPQYSEEARLKREEISG
VKLESIGIYQILSIYSTVASSLVLAIMMAGLSLWMCSNGSLQCRICI

Uncorrected pairwise distance between four H5 amino acid sequences				
	A	B	C	D
H5HT		0.02	0.06	0.08
H5NAVET	0.02		0.07	0.09
H5R65	0.06	0.07		0.04
H5TG	0.08	0.09	0.04	

>Trimeric GCN4-pII motif: KRMKQIEDKIEEILSKIYHIENEIARIKKLIGER

>HHHHHH: 6x histidin tag

>EQKLISEEDL: c-myc tag

>KDEL: ER retention signal tag

>H5TG trimer

DQICIGYHANNSTEQVDTIMEKNVTVTHAQDILEKTHNGKLCDLDGVKPLILRDCSVAGWLLGNPMCDEFINV
PEWSYIVEKANPVNDLCYPGDFNDYEELKHLLSRINHFEKIQIIPKSSWPSHEASLGVSSACPYQGKSSFFRNVVW
LIKKNSTYPTIKRSYNNTNQEDLLVLWGIHHPNDAAEQTKLYQNPTTYISVGTSTLNQRLVPRIATRSKVNGQSG
RMEFFWTILKPNDAINFESNGNFIAPEYAYKIVKKGDSTIMKSELEYGNCNTKCQTPMGAINSSMPFHNIHPLTI
GECPKYVKSNRLVLATGLRNSPQRERRGLFGAIAGFIEGGWQGMVDGWYGYHHSNEQGSGYAADKESTQKAI
DGVTNKVNSIIDKMNTQFEAVGREFNNLERRIENLNKKMEDGFLDVWTYNAELLVLMENERTLDFHDSNVKN
LYDKVRLQLRDNAKELGNGCFEFYHKCDNECMESVRNGTYDYPQYSEEAKLKREEISGVKLEGPKRMKQIEDK
IEEILSKIYHIENEIARIKKLIGERAAAHHHHHHLAAEQKLISEEDLNGSKDEL

>H5HT trimer

DHICIGYHANNSTEQVDTIMEKNVTVTHAQDILEKTHNGKLCDLNGVKPLILKDCSVAGWLLGNPLCDEFINV
PEWSYIVEKPNPANDLCYPGNFNDYEELKHLLSRINHFEKIQIIPKDSWSNHEASLGVSAACSYQGNSSFFRNVV
WLIKKDNAYPTIKKGYNNTNREDLLILWGIHHPNDEAEQTRLYQNPTTYISIGTSTLNQRLVPKIATRSKINGQS
GRIDFFWTILKPNDAIHFESNGNFIAPEYAYKIVKKGDSTIMRSEVEYGNCNTRCQTPIGAINSSMPFHNIHPLTIG
ECPKYVKSNKLVLATGLRNSPQRERRRKRGLFGAIAGFIEGGWQGMVDGWYGYHHSNEQGSGYAADKESTQK
AIDGVTNKVNSIIDKMNTQFEAVGREFNNLERRIENLNKKMEDGFLDVWTYNAELLVLMENERTLDFHDSNV
KNLYDKVRLQLKDNAKELGNGCFEFYHKCNNECMESVRNGTYDYPQYSEEARLKREEISGVKLEGPKRMKQIE
DKIEEILSKIYHIENEIARIKKLIGERAAAHHHHHHLAAEQKLISEEDLNGSKDEL

>Oligomer-TP

DQICIGYHANNSTEQVDTIMEKNVTVTHAQDILEKTHNGKLCDLDGVKPLILRDCSVAGWLLGNPMCDEFINV
PEWSYIVEKANPVNDLCYPGDFNDYEELKHLLSRINHFEKIQIIPKSSWPSHEASLGVSSACPYQGKSSFFRNVVW
LIKKNSTYPTIKRSYNNTNQEDLLVLWGIHHPNDAAEQTKLYQNPTTYISVGTSTLNQRLVPRIATRSKVNGQSG
RMEFFWTILKPNDAINFESNGNFIAPEYAYKIVKKGDSTIMKSELEYGNCNTKCQTPMGAINSSMPFHNIHPLTI

GECPKYVKSNRLVLATGLRNSPQRERRGLFGAIAGFIEGGWQGMVDGWYGYHHSNEQGSGYAADKESTQKAI
DGVTNKVNSIIDKMNTQFEAVGREFNNLERRIENLNKKMEDGFLDVWTYNAELLVLMENERTLDFHDSNVKN
LYDKVRLQLRDNAKELGNGCFEFYHKCDNECMESVRNGTYDYPQYSEEAKLKREEISGVKLEGPKRMKQIEDK
IEEILSKIYHIENEIARIKKLIKPTLYNVSLIMSDTGGTCYAAAHHHHHHLAAEQKLISEEDLNGSKDEL

Tail piece sequence of mouse IgM: KPTLYNVSLIMSDTGGTCY

> Oligomer-HDP

DQICIGYHANNSTEQVDTIMEKNVTVTHAQDILEKTHNGKLCDLDGVKPLILRDCSVAGWLLGNPMCDEFINV
PEWSYIVEKANPVNDLCYPGDFNDYEELKHLLSRINHFEKIQIIPKSSWPSHEASLGVSSACPYQGKSSFFRNVVW
LIKKNSTYPTIKRSYNNTNQEDLLVLWGIHHPNDAAEQTKLYQNPTTYISVGTSTLNQRLVPRIATRSKVNGQSG
RMEFFWTILKPNDAINFESNGNFIAPEYAYKIVKKGDSTIMKSELEYGNCNTKCQTPMGAINSSMPFHNIHPLTI
GECPKYVKSNRLVLATGLRNSPQRERRGLFGAIAGFIEGGWQGMVDGWYGYHHSNEQGSGYAADKESTQKAI
DGVTNKVNSIIDKMNTQFEAVGREFNNLERRIENLNKKMEDGFLDVWTYNAELLVLMENERTLDFHDSNVKN
LYDKVRLQLRDNAKELGNGCFEFYHKCDNECMESVRNGTYDYPQYSEEAKLKREEISGVKLEGPKRMKQIEDK
IEEILSKIYHIENEIARIKKLIGERGGGGSASKLLAGASNWVNQSGSVAQFVFTPSPTQPQTYEVSGNYINNAQGT
GCKGTPYPLSGAYYSGNQIISFSVVWSNASANCQSATGWTGYFDFSGSQAVLKTDWNLAFYSGSTPAIQQGQD
DFMQSVATVSESLLTEAAAHHHHHHLAAEQKLISEEDLNGSKDEL

Homodimer protein (HDP):

SASKLLAGASNWVNQSGSVAQFVFTPSPTQPQTYEVSGNYINNAQGTGCKGTPYPLSGAYYSGNQIISFSVVWS
NASANCQSATGWTGYFDFSGSQAVLKTDWNLAFYSGSTPAIQQGQDDFMQSVATVSESLLTE

>Oligomer-HAP

DQICIGYHANNSTEQVDTIMEKNVTVTHAQDILEKTHNGKLCDLDGVKPLILRDCSVAGWLLGNPMCDEFINV
PEWSYIVEKANPVNDLCYPGDFNDYEELKHLLSRINHFEKIQIIPKSSWPSHEASLGVSSACPYQGKSSFFRNVVW
LIKKNSTYPTIKRSYNNTNQEDLLVLWGIHHPNDAAEQTKLYQNPTTYISVGTSTLNQRLVPRIATRSKVNGQSG
RMEFFWTILKPNDAINFESNGNFIAPEYAYKIVKKGDSTIMKSELEYGNCNTKCQTPMGAINSSMPFHNIHPLTI
GECPKYVKSNRLVLATGLRNSPQRERRGLFGAIAGFIEGGWQGMVDGWYGYHHSNEQGSGYAADKESTQKAI
DGVTNKVNSIIDKMNTQFEAVGREFNNLERRIENLNKKMEDGFLDVWTYNAELLVLMENERTLDFHDSNVKN
LYDKVRLQLRDNAKELGNGCFEFYHKCDNECMESVRNGTYDYPQYSEEAKLKREEISGVKLEGPKRMKQIEDK
IEEILSKIYHIENEIARIKKLIGERGGGGSASELLEDIRKFNEMRKNMDQLKEKINSVLSIRQAAAHHHHHHLAAE
QKLISEEDLNGSKDEL

Homoantiparallel peptide (HAP): SASELLEDIRKFNEMRKNMDQLKEKINSVLSIRQ

>H5HT oligomer-TP

DQICIGYHANNSTEQVDTIMEKNVTVTHAQDILEKTHNGKLCDLDGVKPLILRDCSVAGWLLGNPMCDEFINV
PEWSYIVEKANPVNDLCYPGDFNDYEELKHLLSRINHFEKIQIIPKSSWPSHEASLGVSSACPYQGKSSFFRNVVW
LIKKNSTYPTIKRSYNNTNQEDLLVLWGIHHPNDAAEQTKLYQNPTTYISVGTSTLNQRLVPRIATRSKVNGQSG
RMEFFWTILKPNDAINFESNGNFIAPEYAYKIVKKGDSTIMKSELEYGNCNTKCQTPMGAINSSMPFHNIHPLTI
GECPKYVKSNRLVLATGLRNSPQRERRGLFGAIAGFIEGGWQGMVDGWYGYHHSNEQGSGYAADKESTQKAI
DGVTNKVNSIIDKMNTQFEAVGREFNNLERRIENLNKKMEDGFLDVWTYNAELLVLMENERTLDFHDSNVKN
LYDKVRLQLRDNAKELGNGCFEFYHKCDNECMESVRNGTYDYPQYSEEAKLKREEISGVKLEGPKRMKQIEDK
IEEILSKIYHIENEIARIKKLIKPTLYNVSLIMSDTGGTCYAAAHHHHHHLAAEQKLISEEDLNGSKDEL

Tail piece sequence of mouse IgM: KPTLYNVSLIMSDTGGTCY
